# Supplementary material for: OsUGT88C3 Encodes a UDP-Glycosyltransferase Responsible for Biosynthesis of Malvidin 3-O-Galactoside in Rice
Source: Plants (Basel). 2024 Feb 29;13(5):697. doi: 10.3390/plants13050697 (PMC10934233; doi:10.3390/plants13050697)
Supplement: Supplementary file 1 [file plants-13-00697-s001.zip › Supplemental Figure TIFF/Supplemental Figure with captions.pdf]

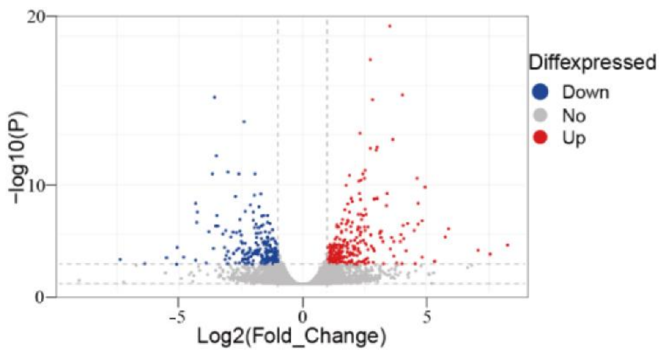

Figure S1. The volcano plots of differentially expressed genes between the top second leaf (L2) and the third leaf (L3) in HN.

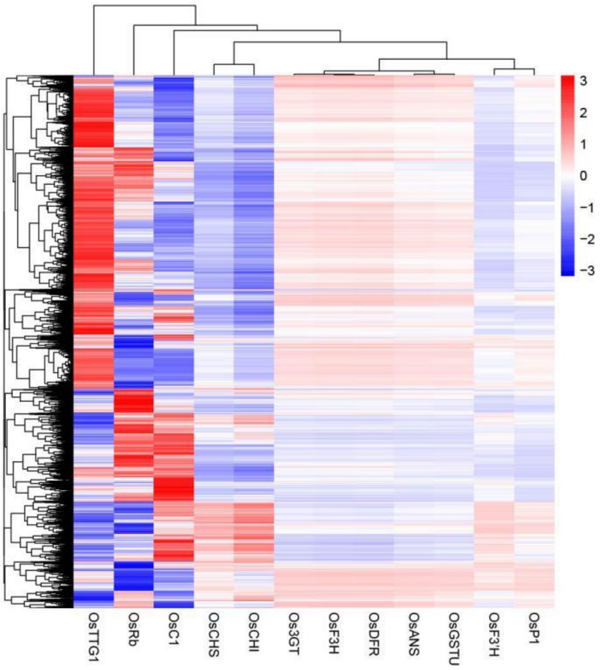

Figure S2. Hierarchical cluster analysis of coexpression correlation coefficients. Hierarchically clustered heatmap of correlation coefficients between expression profiles of all expressed genes detected in the transcriptome sequencing and each ABGs, including OsCHS OsCHI, OsF3H, OsF3′H, OsDFR, OsANS, Os3GT, OsGSTU, OsC1,OsP1, OsRb and OsTTG1.

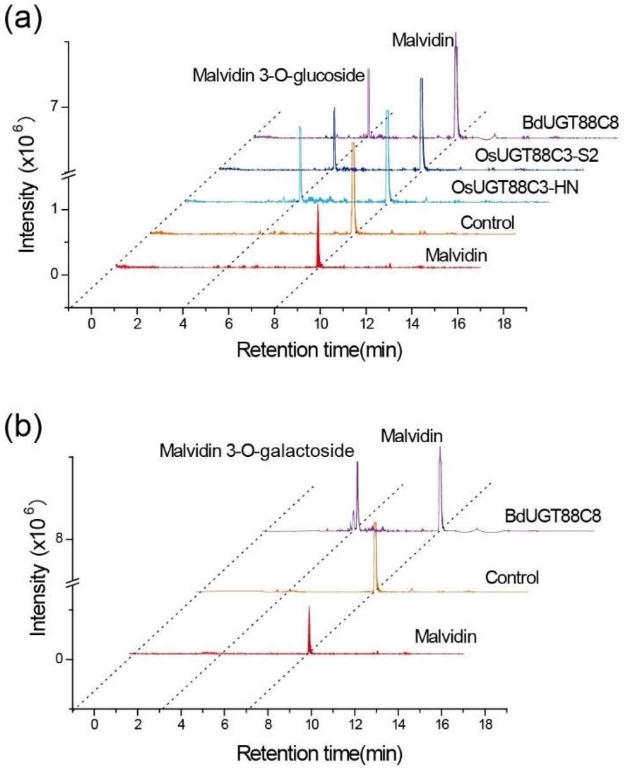

Figure S3. In vitro enzyme assays of OsUGT88C3 and BdUGT88C8. (a) HPLC chromatograms of the in vitro reaction of OsUGT88C3 or BdUGT88C8 with UDP-glucose and malvidin. The data of BdUGT88C8, and OsUGT88C3 from varieties HN (OsUGT88C3-HN) and S2 (OsUGT88C3-S2) are shown. (a) HPLC chromatograms of the in vitro reaction of BdUGT88C8 with UDP-galactose and malvidin.

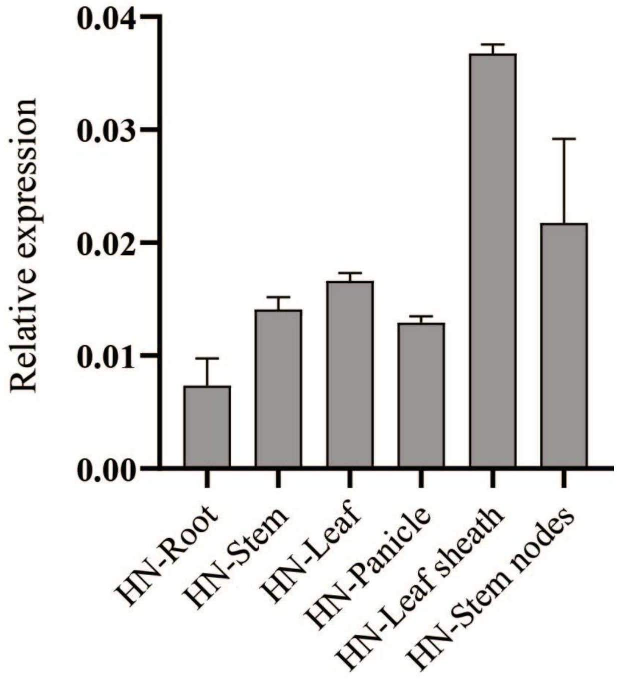

Figure S4. Expression pattern of OsUGT88C3. Total RNA was extracted from roots, stems, leaves, sheaths, panicles and stem nodes, of varieties HN at heading stage. Expression levels of OsUGT88C3 were examined by quantitative reverse transcription-polymerase chain reaction (qRT-PCR). The data are represented as mean ± SD, n = 3.
