# Supplementary figures and images for: OsUGT88C3 Encodes a UDP-Glycosyltransferase Responsible for Biosynthesis of Malvidin 3-O-Galactoside in Rice
Source: Plants (Basel). 2024 Feb 29;13(5):697. doi: 10.3390/plants13050697 (PMC10934233; doi:10.3390/plants13050697)

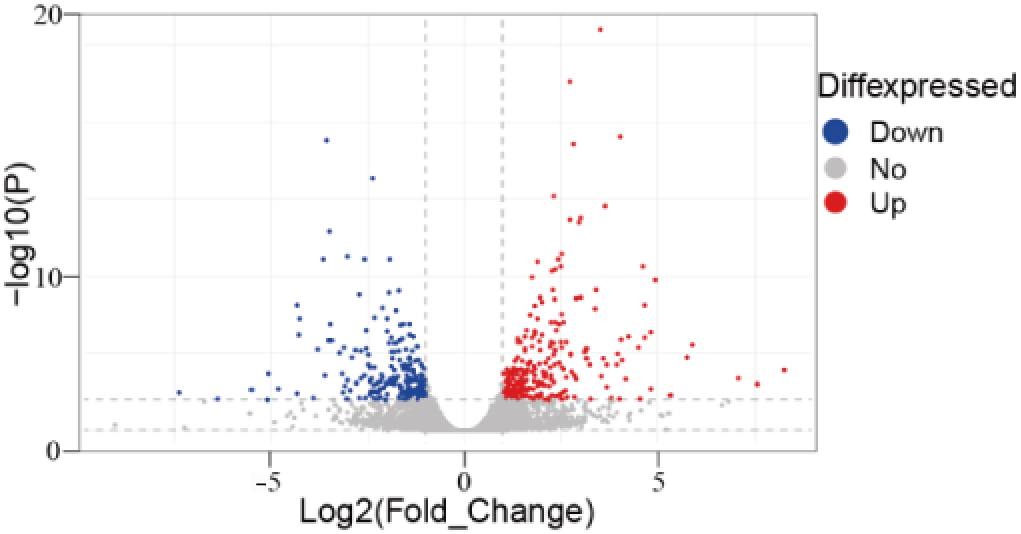

Supplement: Supplementary file 1 [file plants-13-00697-s001.zip › Supplemental Figure TIFF/Figure S1.tif]

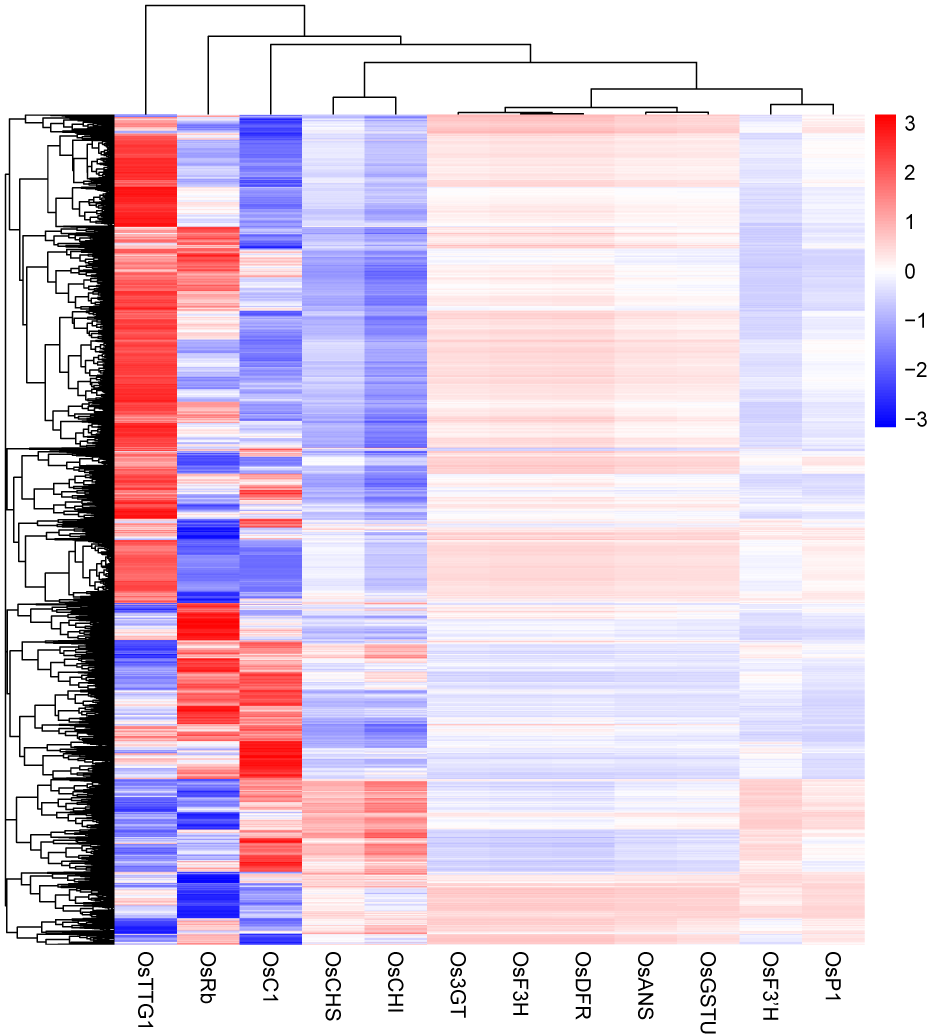

Supplement: Supplementary file 1 [file plants-13-00697-s001.zip › Supplemental Figure TIFF/Figure S2.tif]

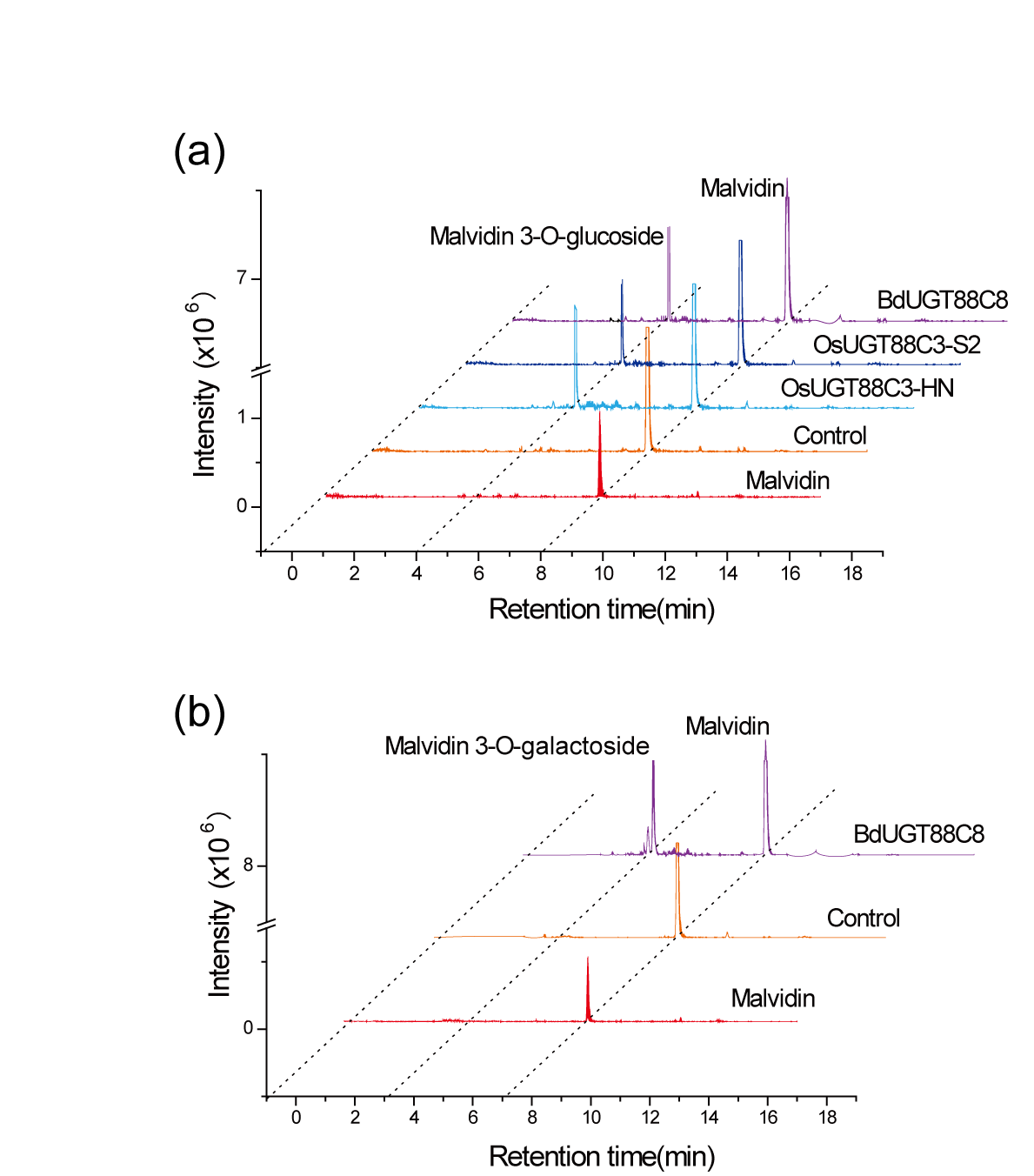

Supplement: Supplementary file 1 [file plants-13-00697-s001.zip › Supplemental Figure TIFF/Figure S3.tif]

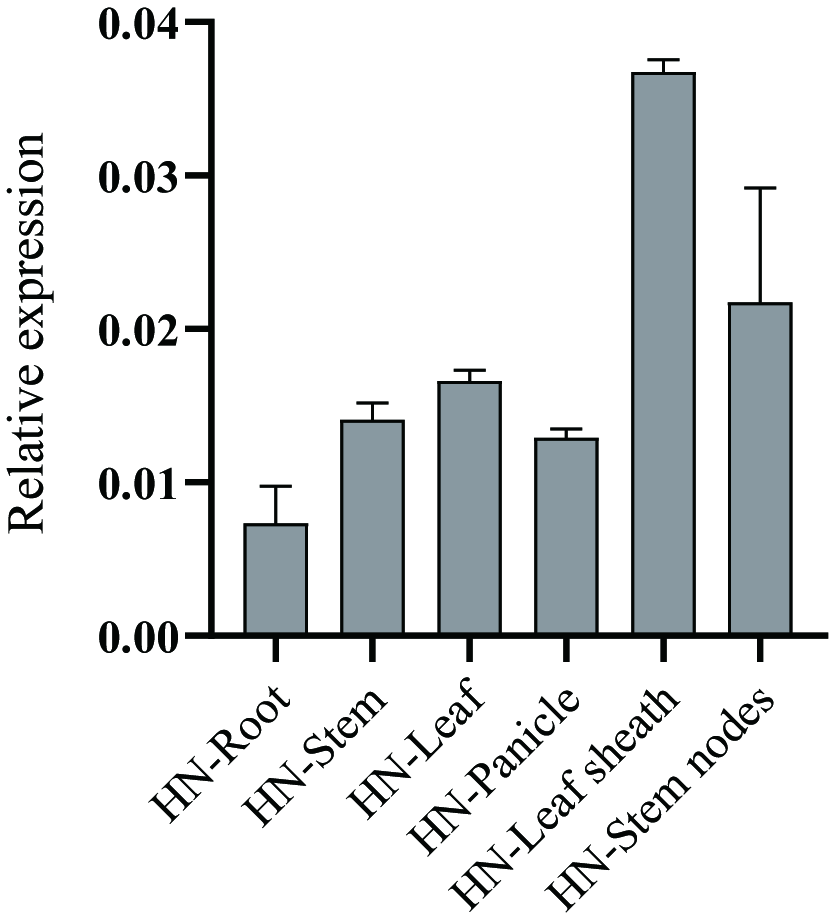

Supplement: Supplementary file 1 [file plants-13-00697-s001.zip › Supplemental Figure TIFF/Figure S4.tif]
